# Haemophagocytic lymphohistiocytosis in critically ill adults: a single-centre retrospective ICU cohort study

**DOI:** 10.3389/fmed.2025.1641659

**Published:** 2025-10-29

**Authors:** Markus Haar, Alina Stadermann, Kevin Roedl, Joseph Tintelnot, Martin Krusche, Fabian Gleibs, Axel Nierhaus, Paymon Ahmadi, Stefan Kluge, Hanno Witte, Dominic Wichmann

**Affiliations:** ^1^Department of Intensive Care Medicine, University Medical Center Hamburg-Eppendorf, Hamburg, Germany; ^2^Department of Hematology and Oncology, Bundeswehrkrankenhaus Ulm, Ulm, Germany; ^3^II. Department of Medicine (Oncology Center), University Medical Center Hamburg-Eppendorf, Hamburg, Germany; ^4^Division of Rheumatology and Systemic Inflammatory Diseases, III. Department of Medicine, University Medical Center Hamburg-Eppendorf, Hamburg, Germany; ^5^Department of Stem Cell Transplantation, University Medical Center Hamburg-Eppendorf, Hamburg, Germany

**Keywords:** HLH, intensive care unit, HScore, haemophagocytic lymphohistiocytosis, critical illness, hyperinflammation, hyperferritiaemia

## Abstract

**Background:**

Haemophagocytic lymphohistiocytosis (HLH) is a life-threatening hyperinflammatory syndrome characterised by uncontrolled immune activation and multi-organ dysfunction. Whilst initially described in paediatric populations, HLH is increasingly recognised in critically ill adults, often triggered by malignancies, infections, or autoimmune diseases.

**Methods:**

This single-centre retrospective study analysed 43 adult patients with HLH admitted to the ICU between 2008 and 2024. Clinical characteristics, laboratory parameters, organ support requirements, and outcomes were assessed. Temporal trends in routine parameters surrounding HLH diagnosis were also evaluated.

**Results:**

The median age was 45 years (IQR: 33–60), and 65% were male. Respiratory failure (62.8%) and sepsis (41.9%) were the leading causes of ICU admission. Disease severity was high, with a median SOFA score of 14 (IQR: 11–17), and ICU mortality reached 65.1%. Invasive mechanical ventilation was required in 83.7% of patients, and continuous renal replacement therapy in 72.1%. Ferritin levels were markedly elevated, with a median peak of 25,045 μg/l (IQR: 12,771–94,586), with 93% of patients exceeding 6,000 μg/l. The median HScore was 245 (IQR: 210–273) and did not differ significantly between survivors and non-survivors (*p* = 0.64).

**Conclusion:**

HLH in adult ICUs carries high mortality and demands extensive organ support. Existing diagnostic scores provide limited bedside guidance, highlighting the need for ICU-specific validation and improved prognostic markers.

## Graphical abstract

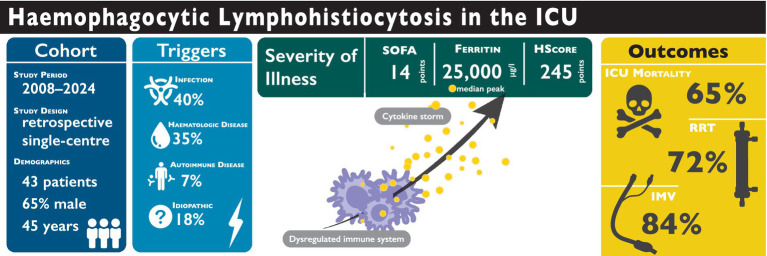

## Introduction

Haemophagocytic lymphohistiocytosis (HLH) is a rare, potentially fatal disorder marked by uncontrolled immune activation and systemic inflammation (1–4). Although initially recognised in paediatric populations (primary), secondary HLH can affect individuals across all age groups and is frequently triggered by infections, malignancies, autoimmune diseases, or immunodeficiencies in adults (4). Its ability to present with non-specific, sepsis-like symptoms contributes to frequent misdiagnosis and delays in appropriate treatment (5, 6). Pathophysiologically, defective granule-mediated cytotoxicity of natural-killer (NK) and CD8^+^ T cells sustains interferon-γ–driven macrophage activation and cytokine storm (4, 7). HLH is classically divided into primary and secondary disease (4): Primary HLH includes perforin-pathway defects (PRF1, UNC13D, STX11, STXBP2, etc.) together with an expanding group of inborn errors of immunity that undermine cytotoxic granule release or inflammasome regulation (4, 8, 9). Whilst primary HLH is most often diagnosed in children, secondary HLH can also occur in paediatric patients, driven by infections, malignancies, or autoimmune disease, similar to adult cases (3, 4, 10). Secondary HLH arises without a monogenic defect when external drivers push immune activation beyond a pathological threshold, with typical precipitants including acute infection, haematologic malignancy, autoimmune flare, and iatrogenic immune activation (e.g., checkpoint inhibitors and CAR-T) (2, 4).

Clinically, HLH presents with non-specific symptoms, including fever, cytopenia, hepatosplenomegaly, and multi-organ dysfunction. Elevated ferritin levels are a hallmark but non-specific finding, necessitating a high index of suspicion, particularly in critically ill patients unresponsive to standard sepsis treatments (3, 11–14).

Although HLH-2004 criteria and the HScore show reasonable diagnostic accuracy in some adult ICU cohorts (11, 12), bedside use is limited by overlap with severe systemic inflammation (e.g., septic shock) (5, 12, 15), reliance on timely bone-marrow examination, and restricted access to specialised assays (16).

The HScore estimates the probability of HLH using a weighted combination of clinical, laboratory, and cytological features—including immunosuppression, fever, cytopenias, organomegaly, ferritin, triglycerides, fibrinogen, AST, and bone marrow haemophagocytosis (11). Whilst scores above 250 indicate a >99% likelihood of HLH, performance in ICU populations is diminished by sepsis-related overlap (6, 12).

A recent multicentre validation of the HLH-2024 criteria in adults demonstrated improved diagnostic feasibility (17). However, data from strictly ICU populations remain limited. Although reports of adult HLH are accumulating (6, 12, 15, 18, 19), awareness across interdisciplinary ICUs remains limited, raising the risk of under-recognition. Detailed descriptions of presentation, disease course, and outcomes in critically ill adults are therefore scarce, underscoring the need for the present study, which also explores predictors of mortality.

## Methods

### Study design and setting

This retrospective observational study was conducted at the Department of Intensive Care Medicine at the University Medical Centre Hamburg-Eppendorf (Germany). The department comprises 12 intensive care units (ICUs) with a total capacity of 120 beds, providing care for critically ill adult patients. Data from patients admitted between 1 January 2008 and 31 January 2024 were included in the analysis.

### Patient identification and data collection

All adult patients admitted to our ICU who were coded with HLH in the hospital’s medical coding system during this period were identified. Records were cross-checked against ICU admission logs. No duplicates were found and no cases were excluded; thus, the screened cohort was identical to the final cohort (*n* = 43). Because our data capture was restricted to our ICU department, HLH cases managed elsewhere in the hospital could not be counted. Most patients were admitted to the ICU following initial management on general wards; however, admission pathways were not systematically recorded in the dataset. A flow diagram is provided in Supplementary Figure S1. After case identification, electronic health record (EHR) data—including ICU-specific variables, comorbidities, and the most likely HLH trigger—were retrieved for analysis.

The HScore was calculated on the day of diagnosis (day 0) to estimate the probability of HLH. The components included the number of cytopenias (affecting one to three lineages), maximum body temperature (°C), ferritin (μg/l), triglycerides (mg/dl), fibrinogen (mg/dl), AST (U/l), presence of underlying immunosuppression, organomegaly, and bone marrow findings suggestive of HLH. Organomegaly was identified from clinical documentation and supplemented by review of available abdominal imaging, including computed tomography (CT) scans and sonograms. Missing values were assigned zero points, consistent with the original scoring methodology.

The comorbidity burden was assessed using the Charlson Comorbidity Index (CCI) (20), whilst disease severity on ICU admission was evaluated using the Simplified Acute Physiology Score (SAPS-II) (21) and the Sequential Organ Failure Assessment (SOFA) (22) score.

### Data processing and statistical analysis

All statistical analyses were conducted using R (version 4.4.1) and RStudio (version 2024.04.2 + 764); data preparation was performed in Python 3.11 with Pandas 2.2.3.

Continuous variables are reported as median (IQR) and compared with the Wilcoxon rank-sum test. Categorical variables are presented as counts (%) and compared with Pearson’s χ^2^ test; Fisher’s exact test was used when any expected cell count was <5.

Organ support therapies—including continuous renal replacement therapy (CRRT), non-invasive ventilation (NIV), invasive mechanical ventilation (IMV), and extracorporeal membrane oxygenation (ECMO)—were analysed with respect to their frequency and duration.

Descriptive analyses were performed for the first 24 h following ICU admission (admission day). To assess temporal trends, clinical and laboratory parameters were aligned to the day of HLH diagnosis (designated as day 0) based on manual review of clinical records. The observation window extended from 2 days prior (day −2) to 6 days following diagnosis (day +6). Within this timeframe, values were aggregated per ICU-day and patient using the median and IQR. For the resulting set of patient-level medians, the cohort median (with IQR) as well as the minimum and maximum were calculated.

Parameters included haemoglobin (g/dl), white blood cell count (10^9^/l), platelet count (10^9^/l), aspartate aminotransferase (AST, U/l), fibrinogen (g/l), lactate dehydrogenase (LDH, U/l), ferritin (μg/l), C-reactive protein (CRP, mg/l), triglycerides (mg/dl), soluble interleukin-2 receptor (sIL-2R, U/l), alveolar-arterial oxygen gradient (AaDO2, mmHg), PaO2/FiO2 ratio (mmHg), lactate (mmol/l), and noradrenaline dose (μg/kg/min). *p*-values < 0.05 were considered statistically significant.

### Ethical considerations

The ethics committee of the Hamburg Chamber of Physicians was informed about the study. Owing to its retrospective design, formal ethical approval was deemed unnecessary (Ethics number: 2023-300397-WF).

## Results

A total of 43 adults with HLH were managed in the ICU (Table 1). Median age was 45 years (IQR: 33–60), and 28 (65%) were male. Median weight and body mass index (BMI) were 75 kg (IQR: 66–83) and 25.0 kg/m^2^ (IQR: 22.0–27.8), respectively. The time from hospital admission to HLH diagnosis was 12 days (IQR 4–25). ICU length of stay was 9 days (IQR: 5–18), which was significantly shorter in non-survivors (8 days [IQR: 3–13] vs. 16 days [IQR: 7–37], *p* = 0.012). Overall, ICU mortality was 65.1% (28/43).

**Table 1:** Baseline characteristics and ICU admission data of patients with HLH stratified by ICU mortality.

Admission day	ICU mortality			*p*-value^b^
	Overall *N* = 43	Survived *N* = 15^a^	Died *N* = 28^a^	
Patient characteristics				
Age	45 (33–60)	44 (29–54)	46 (38–62)	0.40
Sex, female	15 (35)	5 (33)	10 (36)	>0.99
Weight (kg)	75 (66–83)	75 (67–80)	75 (64–87)	0.98
BMI (kg/m^2^)	25.0 (22.0–27.8)	23.3 (21.9–28.2)	26.1 (22.9–27.8)	0.97
ICU LOS (d)	9 (5–18)	16 (7–37)	8 (3–13)	**0.012**
IMV, duration (d)	4 (1–11)	10 (4–29)	2 (1–6)	**0.013**
ICU admission causes				
Respiratory	27 (63)	9 (60)	18 (64)	>0.99
Sepsis/infection	18 (42)	9 (60)	9 (32)	0.15
Oncological/immunological	18 (42)	2 (13)	16 (57)	**0.014**
Haemodynamic	16 (37)	7 (47)	9 (32)	0.54
Renal/metabolic	15 (35)	7 (47)	8 (29)	0.39
Gastrointestinal	12 (28)	2 (13)	10 (36)	0.23
Neurological	11 (26)	5 (33)	6 (21)	0.63
Other	3 (7.0)	2 (13)	1 (3.6)	0.57
Surgical/postoperative	1 (2.3)	0 (0)	1 (3.6)	>0.99
Scores				
SOFA	14 (11–17)	14 (11–15)	14 (11–17)	0.39
SAPS	52 (38–60)	43 (22–50)	57 (44–61)	**0.002**
CCI	1 (0–4)	1 (0–4)	1 (0–4)	0.98
Vitals				
MAP, min (mmHg)	58 (52–62)	56 (52–61)	58 (50–63)	0.86
SpO_2_, min (%)	88 (83–91)	89 (84–91)	88 (75–90)	0.45
Heart rate (bpm)	111 (98–121)	114 (100–120)	111 (95–122)	0.91
Noradrenaline (μg/kg/min)	0.208 (0.069–0.537)	0.135 (0.069–0.268)	0.377 (0.104–0.863)	0.11
Arterial BGA				
PF ratio (mmHg)	231 (158–306)	233 (221–305)	229 (152–315)	0.65
pH	7.41 (7.34–7.44)	7.43 (7.36–7.47)	7.40 (7.33–7.44)	0.13
HCO_3_ (mmol/l)	21.9 (17.7–25.3)	23.4 (20.6–25.9)	21.1 (17.2–24.0)	0.21
pCO_2_ (mmHg)	35 (32–40)	36 (34–38)	34 (31–40)	0.90
Lactate (mmol/l)	3.0 (1.8–4.9)	1.6 (0.9–4.4)	3.3 (2.0–6.8)	0.072

a Median (IQR); *n* (%).

b Wilcoxon rank-sum test for continuous variables; Pearson’s χ^2^ test for categorical variables.

Non-survivors showed a higher illness severity (SAPS II), a greater proportion of oncologic/ immunologic admission causes, and, paradoxically, shorter ICU and ventilation durations—findings consistent with early death rather than reduced support needs. *Reached statistical significance (*p* < 0.05).

HLH, haemophagocytic lymphohistiocytosis; ICU, intensive care unit; IMV, invasive mechanical ventilation; WBC, white blood cell count; Hb, haemoglobin; PLT, platelets; AST, aspartate aminotransferase; ALT, alanine aminotransferase; LDH, lactate dehydrogenase; CRP, C-reactive protein; PCT, procalcitonin; sIL-2R, soluble interleukin-2 receptor; FiO2, fraction of inspired oxygen; PEEP, positive end-expiratory pressure; MAP, mean arterial pressure; SOFA, Sequential Organ Failure Assessment score; SAPS, Simplified Acute Physiology Score; PF ratio, paO2/FiO2 ratio.

Organ support therapies were frequently required (Supplementary Table S1): IMV in 36/43 (83.7%; median 4.0 days, IQR 1.2–10.6), NIV in 22/43 (51.2%; 0.9 days, IQR 0.1–3.0), CRRT in 31/43 (72.1%; 3.3 days, IQR 0.6–9.8), and venovenous ECMO in 3/43 (7.0%; 4.6 days, IQR 1.3–5.4).

### Admission causes and clinical characteristics

The primary reasons for ICU admission included respiratory failure in 27 patients (62.8%), sepsis or infection in 18 patients (41.9%), haemodynamic instability in 16 patients (37.2%), and renal or metabolic complications in 15 patients (34.9%; see Table 1).

The median SOFA score on the admission day was 14 (IQR: 11–17), whilst the median SAPS-II was 52 (IQR: 38–60). The CCI indicated a median score of 1 (IQR: 0–4). Although no significant differences were found between survivors and non-survivors for SOFA scores (14 [IQR: 11–15] vs. 13.5 [IQR: 11–17], *p* = 0.39) or the CCI (1 [IQR: 0–4] vs. 1 [IQR: 0–4], *p* = 0.98), non-survivors had significantly higher SAPS-II scores (57 [IQR: 44–61] vs. 43 [IQR: 22–50], *p* = 0.002).

Routine (median) arterial blood gas analysis (BGA) parameters on the ICU admission day revealed no statistically significant differences between survivors and non-survivors, although the elevation in lactate levels bordered on statistical significance (1.6 [IQR: 0.9–4.4] vs. 3.3 mmol/l [IQR: 2.0–6.8], *p* = 0.072). Noradrenaline was administered to 25 (58.1%) patients on the day of ICU admission, with non-survivors receiving a higher median dose (0.38 μg/kg/min [IQR: 0.10–0.86]) compared to survivors (0.14 μg/kg/min [IQR: 0.07–0.27]), although this difference was not statistically significant (*p* = 0.11). Consult Table 1 for further details.

### HScore and clinical characteristics of HLH

At diagnosis (day 0), the median HScore was 245 (IQR: 210–273), with no observed difference between ICU survivors and non-survivors (228.0 [IQR: 208–278] vs. 249.0 [IQR: 212.5–272.5], *p* = 0.64; see Supplementary Table S2). The majority of patients (93%) had ferritin levels exceeding 6,000 μg/l, and 67% met the fibrinogen criterion (≤ 2.5 g/l), 58% the triglyceride criterion (> 354 mg/dl), and 98% the AST criterion (≥ 30 U/l). Three-lineage cytopenia occurred in 54%, organomegaly in 79%, and haemophagocytosis in 15 of 30 bone-marrow aspirates (50%).

Because several potentially informative markers fall outside the HScore, we also reviewed laboratory values across the interval −2 to +6 days around diagnosis (Supplementary Table S4). Ferritin remained markedly elevated and was higher in non-survivors than survivors (28,060 vs. 14,154 μg/l; *p* = 0.039). Soluble interleukin-2 receptor (sIL-2R) showed a similar outcome gradient—overall median 8,429 U/ml, rising to 28,024 U/ml in non-survivors vs. 6,942 U/ml in survivors (*p* = 0.041)—whereas fibrinogen, triglycerides, AST, and IL-6 did not differ significantly between groups.

Aside from idiopathic cases (28%), the most frequently identified triggers for HLH were Epstein–Barr virus infection (14%), human immunodeficiency virus infection (12%), and haematologic malignancies—including lymphoma (23%), leukaemia (9.3%), and myelodysplastic syndrome (2.3%). Other less common triggers included dermatomyositis, human herpesvirus 8, herpes simplex virus, and vasculitis. Further details can be found in Tables 2a, 2b.

**Table 2a:** Trigger categories in the study cohort.

	ICU mortality		
Characteristic	Overall *N* = 43^a^	Survived *N* = 15^a^	Died *N* = 28^a^
Trigger category			
Bacterial	3 (7.0)	2 (13)	1 (3.6)
Haematologic	15 (35)	6 (40)	9 (32)
Idiopathic	8 (19)	3 (20)	5 (18)
Rheumatic	3 (7.0)	2 (13)	1 (3.6)
Viral	14 (33)	2 (13)	12 (43)

a *n* (%).

Haematologic disease and viral infection were the two most frequent categories overall; viral triggers predominated amongst non-survivors, whereas haematologic triggers were similarly represented in both outcome groups.

ICU, intensive care unit.

**Table 2b:** Specific aetiologic triggers identified.

	ICU mortality		
Characteristic	Overall *N* = 43^a^	Survived *N* = 15^a^	Died *N* = 28^a^
Trigger			
Dermatomyositis	2 (4.7)	1 (6.7)	1 (3.6)
EBV	6 (14)	1 (6.7)	5 (18)
HHV-8	1 (2.3)	0 (0)	1 (3.6)
HIV	5 (12)	1 (6.7)	4 (14)
HSV	1 (2.3)	0 (0)	1 (3.6)
Idiopathic	12 (28)	5 (33)	7 (25)
Leukaemia	4 (9.3)	1 (6.7)	3 (11)
Lymphoma	10 (23)	5 (33)	5 (18)
MDS	1 (2.3)	0 (0)	1 (3.6)
Vasculitis	1 (2.3)	1 (6.7)	0 (0)

a *n* (%).

Concrete infectious agents (notably EBV, HIV, HSV, HHV-8) and malignant or rheumatic conditions underlying each HLH episode, complementing the broader categories shown in Table 2a. For HIV, all cases were pre-existing diagnoses, and HLH was consistently triggered by opportunistic infections.

ICU, intensive care unit; EBV, Epstein-Barr virus; HIV, human immunodeficiency virus; HSV, herpes simplex virus; MDS, myelodysplastic syndrome.

**Figure 1:**
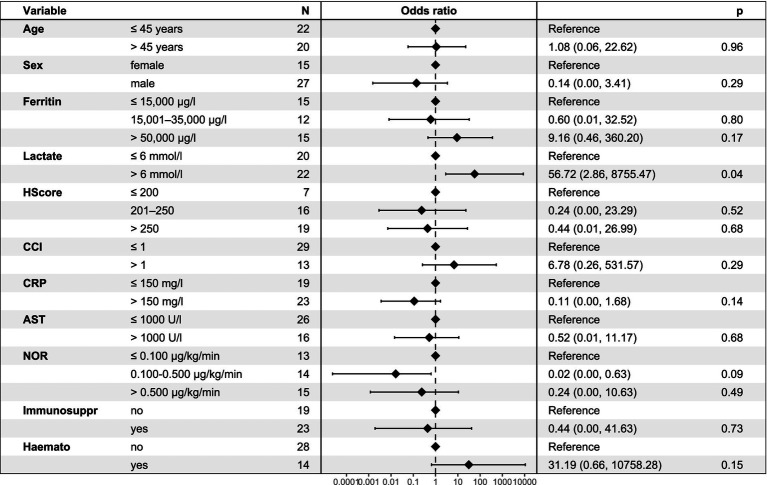
Multivariate logistic regression model for ICU mortality in patients with HLH. Odds ratios (OR) with 95% confidence intervals are shown for selected clinical and laboratory parameters associated with ICU mortality. Elevated lactate levels (>6 mmol/l) were significantly associated with ICU mortality (*p* = 0.04). HLH, haemophagocytic lymphohistiocytosis; CCI, Charlson Comorbidity Index; CRP, C-reactive protein; AST, aspartate aminotransferase; NOR, noradrenaline; Immunosuppr, Immunosuppression; Haemato, Haematologic disease; OR, odds ratio; CI, confidence interval.

In an exploratory multivariable logistic regression model (see Figure 1), a lactate level > 6 mmol/l was the only covariate that independently predicted ICU mortality (OR 16.1, 95%-CI: 1.76–393.9, *p* = 0.03). No significant associations were found for age, sex, HScore, CCI, CRP, AST, ferritin, vasopressor requirements, immunosuppression, or haematologic disease.

### Immunosuppressive therapy administered in the ICU

Immunosuppressive therapy varied across the cohort. The combinations of agents are shown in Supplementary Figure S2, whilst the frequencies of individual therapies administered during the ICU stay are summarised in Table 3.

**Table 3:** Immunosuppressive therapies administered during ICU stay.

Characteristic	*N* = 43^a^
Any steroid	42 (98%)
Dexamethasone	30 (70%)
Prednisolone	30 (70%)
Hydrocortisone	22 (51%)
Etoposide	14 (33%)
Anakinra	13 (30%)
Tocilizumab	4 (9.3%)
Ciclosporin A	3 (7.0%)

a *n* (%).

Shown are therapies actually given in the ICU and their frequencies. Pre-ICU treatments and timing of initiation were not systematically recorded, and no institutional treatment algorithm existed. Data reflect individual treatment decisions rather than a standardised protocol.

### Temporal dynamics of clinical parameters

**Figure 2:**
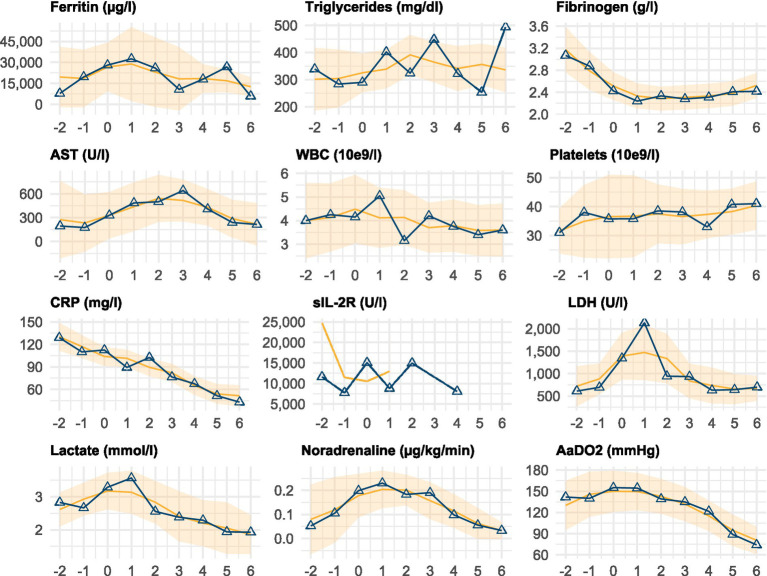
Temporal trends of key clinical and laboratory parameters. Temporal progression of selected clinical and laboratory parameters relative to the day of HLH diagnosis or relapse (day 0). Each subplot displays the median value per day (solid line with markers) and a smoothed trend (running median ± standard error). The data are shown from 2 days before (−2) to 6 days after (+6) diagnosis. The smoother line includes values beyond this window to stabilise edge estimates. Supplementary Table S3 provides the corresponding numerical data. HLH, haemophagocytic lymphohistiocytosis; AST, aspartate aminotransferase; WBC, white blood cell count; CRP, C-reactive protein; sIL-2R, soluble interleukin-2 receptor; LDH, lactate dehydrogenase; AaDO2, alveolar-arterial oxygen difference.

Several parameters exhibited distinct temporal trends around the time of HLH diagnosis (day 0), as outlined in Figure 2 and Supplementary Table S3. White blood cell counts remained low but stable, whilst platelet counts showed a slight increase from a median of 31 × 10^9^/l on day −2 to 41 × 10^9^/l on day +6.

Ferritin levels peaked on day +1 (median 32,528 μg/l [IQR: 21,435–79,516]) and gradually declined thereafter. Similarly, LDH and AST levels increased around diagnosis, with LDH peaking on day +1 (2,131 U/l [IQR: 907–3,457]) and AST on day +3 (642 U/l [IQR: 103–1,335]), followed by gradual decreases. Fibrinogen levels decreased prior to diagnosis but showed modest recovery post-diagnosis.

Vital signs reflected the severity of illness, with mean arterial pressure (MAP) remaining relatively stable (median 75 mmHg [IQR: 71–81]) but noradrenaline requirements peaking around day 1 (0.230 μg/kg/min [IQR: 0.050–0.607]) before declining. Oxygenation parameters, including the alveolar-arterial oxygen difference (AaDO2), remained impaired but showed steady improvement from day +2 onward.

The levels of inflammatory markers, such as CRP and PCT, peaked prior to diagnosis and gradually decreased post-treatment initiation. CRP levels decreased from 129 mg/L (IQR: 114–179) on day −2 to 43 mg/L (IQR: 19–124) on day +6.

In 4 of 43 patients (9.3%), HLH was diagnosed more than 24 h before ICU admission. In these cases, the diagnostic reference point (day 0) preceded ICU admission by a median of 2.5 days (IQR: −6.75 to −2.0), and thus the temporal trends partially reflect pre-ICU disease progression.

## Discussion

Our analysis of 43 adult ICU patients with HLH revealed a high disease burden and 65% ICU mortality. The median age was 45 years—substantially lower than in previously reported ICU cohorts (6, 12, 18)—with respiratory failure (62.8%) and sepsis or infection (41.9%) being the most common causes of admission. The severity of illness was reflected by a median SOFA score of 14 at admission and a high demand for organ support. IMV was required in 83.7% of patients, notably exceeding the 53–59% reported in prior studies (6, 15, 19), whilst RRT was required in 72.1%, comparable only to the cohort described by Knaak et al. (12) Survivors were ventilated longer, presumably reflecting early death amongst non-survivors rather than because prolonged support was beneficial. At presentation (see Table 1), a SOFA of 14 and SAPS II of 52 imply a predicted mortality ≥ 70–90%, well above general-ICU estimates for the same scores (45–55%), underscoring the rapidly progressive nature of HLH (23, 24).

In the subgroup of patients with HIV (12%), HLH was consistently triggered by opportunistic infections. All were previously known HIV cases, and no new diagnoses occurred during the study period. Antiretroviral treatment status was not systematically available. Amongst malignancy-associated HLH, mortality was 60%, consistent with prior reports (4). Interestingly, Löfstedt et al. recently described an increasing incidence of malignancy-associated HLH in their population-based study from Sweden, with rates rising from 0.026 per 100,000 adults (1997–2007) to 0.45 per 100,000 adults (2012–2018). In their cohort, malignancy-associated HLH was identified in 0.6% of haematological malignancies, with the highest proportion amongst young males (2.5%), despite better early survival, 2-year mortality remained 75% (25). In our series, all malignancy-associated HLH occurred in patients with a pre-existing malignancy under treatment. The majority had haematologic neoplasms (lymphoma, leukaemia, or myelodysplastic syndrome). Two patients had local solid tumours, whilst none had metastatic solid cancer.

In our multivariable logistic regression model (see Figure 1), a lactate concentration > 6 mmol/l was the only independent predictor of ICU death; age, sex, HScore, CCI, CRP, AST, ferritin, vasopressor use, immunosuppression, and haematologic disease were not significant. Given the model’s limited power and the heterogeneity of our cohort, these results should be interpreted with caution; however, they support the relevance of routine bedside parameters for early risk stratification.

The HLH-2004 diagnostic criteria remain widely used but were originally developed for paediatric populations (2, 3, 26). Because diagnosis requires ≥ 5 of 8 criteria (27), many ICU patients display suggestive features without crossing the threshold (6).

In initial studies, the HScore appeared robust (cutoff 169: sensitivity 93%, specificity 86%) (11), but those numbers stem from non-ICU cohorts and likely overestimate performance in critical care, where sepsis mimics many score components. A recent multicentre study in adult cohorts (17), including ICU patients, reported that lowering the HLH-2004 threshold to four items yielded the best diagnostic accuracy, whereas the revised HLH-2004 and the HScore ≥169 performed slightly less well. In our series, the median HScore was 245 (IQR 210–273), and—as expected for a tool built for diagnosis rather than prognosis—it did not differ significantly between survivors and non-survivors (median 228 vs. 249, *p* = 0.64; see Supplementary Table S2). We emphasise that any modification of existing cutoffs requires validation against matched ICU non-HLH controls, as sepsis and other hyperinflammatory states share many overlapping features (Table 4). To provide practical guidance, we outline a pragmatic diagnostic pathway tailored for ICU settings (Box 1).

**Table 4:** Differential diagnosis of HLH vs. related ICU syndromes.

Feature/test	HLH (typical)	Sepsis/septic shock	Multi-organ failure (non-inflammatory)	MIS	ICU implication
Fever	Often present, but may be blunted in ICU (antipyretics, sedation, CRRT)	Variable	Variable/absent	Common	Do not rely on fever alone in ICU
Cytopenias	Bi−/tri-lineage common	Thrombocytopenia and anaemia common	Anaemia common; others variable	Variable	Pattern/depth supports HLH but not specific
Ferritin	Markedly ↑, frequently >6,000–10,000 μg/l	↑ Possible, usually lower range	Variable	↑	Extreme ferritin raises suspicion but is not pathognomonic
Fibrinogen	↓ (≤2.5 g/l)	↓/Normal	Variable	Variable	Falling fibrinogen supports HLH but is not specific
Triglycerides	↑	↑ Possible	Variable	Variable	Adds weight to HLH probability
sIL-2R	↑↑	Normal/mild ↑	Not typical	Variable	May add specificity for HLH where available
CXCL9 (IFN-γ signature)	↑ (esp. EBV-HLH)	↑ in IFN-γ sepsis endotype (subset)	Not typical	Variable	Potential discriminator in EBV-HLH
Bone marrow	Haemophagocytosis possible; limited sensitivity; not required	Non-specific	Non-specific	Variable	Feasibility limits in unstable patients
Organomegaly	Hepatosplenomegaly common (esp. splenomegaly)	Variable	Uncommon	Variable	Supports HLH suspicion
Clinical course	Rapid hyperinflammatory deterioration	Shock with infectious focus	Progressive dysfunction without systemic hyperinflammation	Post-infectious hyperinflammation	Trajectory helps frame urgency and work-up
Management focus	Treat trigger + consider immunomodulation with haematology input	Source control + antimicrobials + organ support	Organ support; treat precipitant	Immunomodulation + treat trigger	Early haematology involvement; do not delay infection management

Key clinical features, laboratory tests, and implications for ICU management. Fever may be blunted by ICU interventions, including CRRT (40). Cytopenias are frequent in critical illness (thrombocytopenia, anaemia, and leukopenia) and therefore lack specificity for HLH (36–39). Ferritin can be extreme in HLH but also elevates in other hyperinflammatory states; specificity improves at very high levels yet remains imperfect (13, 15, 28, 29). Hypofibrinogenaemia and hypertriglyceridaemia are established HLH criteria (HLH-2004; HScore) (11, 27). sIL-2R and CXCL9 may increase diagnostic confidence for HLH and EBV-HLH, whilst an IFN-γ–high sepsis endotype also shows CXCL9 elevation (45–47). Bone-marrow haemophagocytosis has limited sensitivity in ICU populations (18). Organomegaly supports HLH but is not exclusive (2). Multisystem inflammatory syndrome (MIS) can mimic HLH and has been described in the context of recent SARS-CoV-2 infection in children (MIS-C) as well as adults (MIS-A) (48).

HLH, haemophagocytic lymphohistiocytosis; MIS, multisystem inflammatory syndrome; CRRT, continuous renal replacement therapy; sIL-2R, soluble interleukin-2 receptor; EBV, Epstein–Barr virus; IFN-γ, interferon-gamma.

**Box 1 Pragmatic ICU diagnostic pathway for suspected HLH**

Trigger to suspect HLH

- Ferritin > 6,000 μg/l with unexplained hyperinflammation,
- plus ≥ 1 of: new cytopenias (≥ 2 lineages), hypofibrinogenaemia, severe transaminase elevation, splenomegaly, or refractory fever/shock **not otherwise explained**

Immediate steps (0–6 h)

- Urgent haematology consult.
- Core laboratory tests: ferritin, triglycerides, fibrinogen, AST/ALT, LDH, CBC with differential, blood film
- Microbiological sampling: cultures and viral PCRs (EBV, CMV, HIV, Adenovirus)

Early adjuncts (6–24 h)

- Soluble IL-2 receptor (sIL-2R) if available
- Bone-marrow aspirate/trephine if feasible; do not delay treatment in unstable patients

Parallel management

- Search for underlying trigger (malignancy, infection, autoimmunity)
- Provide full supportive ICU care and infection management
- Consider immunomodulation per haematology guidance

Daily reassessment

- Re-evaluate likelihood of HLH with accumulating data
- Escalate diagnostics and therapy as appropriate

Proposed pragmatic diagnostic pathway for suspected HLH in critically ill adults.

Structured steps for recognition and early management of haemophagocytic lymphohistiocytosis (HLH) in the ICU. The pathway is intended as practical bedside guidance and does not replace specialist haematology input or prospective validation.

HLH, haemophagocytic lymphohistiocytosis; ICU, intensive care unit; CBC, complete blood count; AST, aspartate aminotransferase; ALT, alanine aminotransferase; LDH, lactate dehydrogenase; EBV, Epstein–Barr virus; CMV, cytomegalovirus; sIL-2R, soluble interleukin-2 receptor.

Hyperferritinaemia, a hallmark of HLH, is frequently observed in critically ill ICU patients without HLH, often as part of the acute phase response (15, 28, 29). In a 12-year ICU cohort study, 2,623 ICU patients had ferritin ≥ 500 μg/l, but only 1.5% of them were ultimately diagnosed with HLH. Amongst those with HLH, peak ferritin concentrations were markedly higher than septic or septic-shock controls (median 31,674 vs. 1,545 and 1,448 μg/l, *p* < 0.001) (29). As highlighted by Liedgens et al., only 17% of patients with ferritin levels >50,000 μg/l were ultimately diagnosed with HLH, illustrating the poor specificity of even extreme hyperferritinaemia in ICU settings (11, 15). Mild-to-moderate transaminase rises are equally non-specific. Ischaemic hepatitis (“shock liver”) affects up to 13.4% of ICU admissions (30, 31), and AST/ALT elevations may also stem from drug-induced liver injury (32) heart failure (33, 34), rhabdomyolysis (31), acute or chronic liver failure (31), or parenteral nutrition-associated liver disease (PNALD) (35). Cytopenias are ubiquitous in critical illness: thrombocytopenia appears in 20–50% of patients (36, 37), and anaemia develops in nearly all long-stay ICU patients (38); leukopenia or neutropenia may follow sepsis or myelosuppressive therapy (39). Fever—a cornerstone HLH sign—was present in only 39.5% of our cases. Antipyretics, deep sedation, neuromuscular blockade, and heat-dissipating treatments (e.g., CRRT and large-volume infusions) routinely depress temperature; in a 587-patient CRRT cohort, temperature curves were identical in infected and non-infected patients (40), highlighting the reduced sensitivity of this sign.

In our cohort, bone marrow aspirates were performed in only 30 of 43 patients (69.8%), and haemophagocytosis was identified in just 15 of those (50%), reflecting limited yield and possible difficulty of sampling unstable patients (see Supplementary Table S2). Such limitations might delay diagnosis and contribute to under-recognition of HLH in the ICU setting, as haemophagocytosis may not be required for diagnosis (18).

Published data demonstrate that interferon-γ–inducible chemokines, particularly CXCL9, are significantly higher in HLH compared with sepsis, whereas IL-6 is elevated in both conditions but typically reaches higher levels in sepsis (41). In EBV-associated HLH, additional studies have shown disproportionately high IFN-γ and IL-10 with low IL-6, yielding IFN-γ/IL-6 and IL-10/IL-6 ratios that robustly differentiate EBV-HLH from sepsis (42). More recently, immune profiling has confirmed that acute EBV infection induces CXCL9/10/11 and IFN-γ–driven T-cell activation, providing a mechanistic link between EBV biology and the cytokine storm underlying HLH (43). With respect to presepsin (sCD14-ST), evidence in HLH is sparse. A small series in malignancy-associated HLH reported that presepsin levels alone were not prognostic, but a composite of presepsin and sIL-2R was associated with 90-day mortality (44). These assays were not available in our ICU, and their diagnostic role requires prospective validation before incorporation into routine panels. Because HLH shares many features with sepsis, septic shock, non-inflammatory multi-organ failure, and multisystem inflammatory syndrome (MIS), we provide a comparative differential diagnosis table (Table 4) highlighting overlapping findings and key discriminating tests relevant for ICU practise.

Future multicentre studies should combine refined clinical scores with rigorously validated biomarker panels to accelerate diagnosis, guide immunomodulatory therapy, and improve outcomes in ICU-HLH.

## Limitations

Our study has several limitations. First, the retrospective design may have introduced selection and information biases, particularly regarding data completeness and case identification. Clinical recognition and coding practices may also have evolved between 2008 and 2024, which could have affected case capture. In addition, conditions such as seronegative rheumatoid arthritis, immune reconstitution in HIV, or bone marrow reconstitution were likely misclassified in some cases. Information on the timing of bone marrow aspiration was not systematically recorded, and in several cases, only the pathology report date or the ICU discharge summary documented positive findings. Consequently, we could not determine a reliable interval between diagnosis and sampling. The limited diagnostic yield (haemophagocytosis in 15/30 aspirates) may therefore partly reflect delayed or post-treatment sampling.

Second, the single-centre nature limits the generalisability of our findings, as HLH management strategies, diagnostic approaches, and ICU admission criteria may vary across institutions. Third, the reliance on the clinical day of diagnosis to align temporal data. Whilst this is clinically relevant, the timing of HLH diagnosis is subject to physician discretion and available diagnostics, potentially misaligning disease onset with laboratory and clinical trends. This could affect the interpretation of disease progression and treatment responses. Fourth, pre-ICU treatments for HLH could not be assessed systematically, preventing us from fully evaluating their impact on ICU outcomes. Finally, functional assays for cytotoxicity (e.g., perforin expression) and genetic testing for inborn errors of immunity (next-generation sequencing panels, whole-exome sequencing, or whole-genome sequencing) were not systematically performed or recorded in our cohort and thus were not accessible for analysis. As these investigations are not part of routine ICU diagnostics, we cannot exclude that rare, previously unrecognised inborn error of immunity contributed to susceptibility in individual cases.

## Conclusion

This study highlights the substantial disease burden and high mortality (65%) and organ-support requirements associated with HLH in adult ICU patients. Although nearly all cases met conventional HLH-2004 criteria and far exceeded the diagnostic HScore threshold, many of the individual variables—fever, cytopenias, transaminase elevations, hyperferritinaemia—also occur in sepsis and multi-organ failure, blurring the distinction between HLH and other hyper-inflammatory states in critical care. A lactate concentration > 6 mmol/l emerged as the sole bedside variable independently linked to ICU mortality in our exploratory model, but the HScore itself, designed for diagnosis rather than prognosis, showed no survival discrimination.

In critically ill adults, the individual components of current HLH scores frequently overlap with sepsis-related abnormalities, complicating bedside interpretation. To clarify the real-world performance of these tools in the ICU—and to determine whether adjunctive immunologic markers add value—larger, prospective cohorts that include both HLH and non-HLH patients are needed.

## Data Availability

The datasets presented in this article are not readily available because data protection regulations. Requests to access the datasets should be directed to m.haar@uke.de.

## Glossary

AaDO2: Alveolar–arterial oxygen difference
ALT: Alanine aminotransferase
AST: Aspartate aminotransferase
BGA: Blood gas analysis
CCI: Charlson Comorbidity Index
CRP: C-reactive protein
CRRT: Continuous renal replacement therapy
CSA: Ciclosporin A
ECMO: Extracorporeal membrane oxygenation
EBV: Epstein–Barr virus
FiO2: Fraction of inspired oxygen
Hb: Haemoglobin
HIV: Human immunodeficiency virus
HLH: Haemophagocytic lymphohistiocytosis
HSV: Herpes simplex virus
ICU: Intensive care unit
IMV: Invasive mechanical ventilation
IQR: Interquartile range
LDH: Lactate dehydrogenase
MAP: Mean arterial pressure
MDS: Myelodysplastic syndrome
MIS: Multisystem inflammatory syndrome
NIV: Non-invasive ventilation
OR: Odds ratio
PCT: Procalcitonin
PEEP: Positive end-expiratory pressure
PLT: Platelets
SAPS-II: Simplified Acute Physiology Score II
sIL-2R: Soluble interleukin-2 receptor
SOFA: Sequential Organ Failure Assessment
WBC: White blood cell count
