# Supplementary material for: Haemophagocytic lymphohistiocytosis in critically ill adults: a single-centre retrospective ICU cohort study
Source: Front Med (Lausanne). 2025 Oct 29;12:1641659. doi: 10.3389/fmed.2025.1641659 (PMC12605457; doi:10.3389/fmed.2025.1641659)
Supplement: Supplementary file 1 [file Data_Sheet_1.pdf]

doi: 10.3389/fmed.2025.1641659

Supplement

Table S1

Characteristic	Overall<br>N = 43[1]	ICU Mortality<br>Survived<br>N = 15[1]	 Died<br>N = 28[1]	p-value[2]
**NIV**	22 (51.2)	6 (40.0)	16 (57.1)	0.28
**IMV**	36 (83.7)	11 (73.3)	25 (89.3)	0.22
**CRRT**	31 (72.1)	8 (53.3)	23 (82.1)	0.074
**ECMO**	3 (7.0)	1 (6.7)	2 (7.1)	>0.99

[1] n (%)

[2] Pearson's Chi-squared test; Fisher's exact test

Supplementary Table 1: Requirement of organ support stratified by ICU mortality

Frequency and percentage of patients receiving NIV, IMV, CRRT, and ECMO. Data are shown overall and stratified by ICU mortality.

Abbreviations: HLH = haemophagocytic lymphohistiocytosis, NIV = non-invasive ventilation, IMV = invasive mechanical ventilation, CRRT = continuous renal replacement therapy, ECMO = extracorporeal membrane oxygenation.

Table S2

Characteristic	Overall N = 43[1]	ICU Mortality: Survived N = 15[1]	ICU Mortality: Died N = 28[1]	p-value[2]
**Temperature, °C**				0.26
< 38.4	26 (60.5)	10 (66.7)	16 (57.1)	
38.4-39.4	12 (27.9)	5 (33.3)	7 (25.0)	
> 39.4	5 (11.6)	0 (0.0)	5 (17.9)	
**Cytopenias, n**				0.25
1 lineage	1 (2.3)	0 (0.0)	1 (3.6)	
2 lineages	19 (44.2)	9 (60.0)	10 (35.7)	
3 lineages	23 (53.5)	6 (40.0)	17 (60.7)	
**Ferritin (µg/l)**				0.43
< 2000	1 (2.3)	1 (6.7)	0 (0.0)	
2000-6000	2 (4.7)	1 (6.7)	1 (3.6)	
> 6000	40 (93.0)	13 (86.7)	27 (96.4)	
**Triglycerides, mg/dl**				0.32
< 132.7	3 (7.0)	0 (0.0)	3 (10.7)	
132.7-354	15 (34.9)	7 (46.7)	8 (28.6)	
> 354	25 (58.1)	8 (53.3)	17 (60.7)	
**Fibrinogen, mg/dl**				0.51
≤ 2.5	29 (67.4)	9 (60.0)	20 (71.4)	
> 2.5	14 (32.6)	6 (40.0)	8 (28.6)	
**AST, U/l**				0.35
< 30	1 (2.3)	1 (6.7)	0 (0.0)	
≥ 30	42 (97.7)	14 (93.3)	28 (100.0)	
**Organomegaly**				0.10
liver	9 (20.9)	4 (26.7)	5 (17.9)	
spleen	8 (18.6)	1 (6.7)	7 (25.0)	
both	17 (39.5)	9 (60.0)	8 (28.6)	
none	9 (20.9)	1 (6.7)	8 (28.6)	
**Bone marrow suggestive of HLH**				0.70
No	15 (50.0)	6 (54.5)	9 (47.4)	
Yes	15 (50.0)	5 (45.5)	10 (52.6)	
Unknown	13	4	9	
**Immunosuppression**				0.81
No	19 (44.2)	7 (46.7)	12 (42.9)	
Yes	24 (55.8)	8 (53.3)	16 (57.1)	
**H-Score, points**	245.0 (210.0 – 273.0)	228.0 (208.0 – 278.0)	249.0 (212.5 – 272.5)	0.64

[1] n (%); Median (IQR)

[2] Fisher's exact test; Pearson's Chi-squared test; Wilcoxon rank sum test

Supplementary Table 2. Clinical and laboratory parameters included in the HScore as observed in the cohort of patients with HLH

The HScore is a validated diagnostic tool for HLH, with a total score ranging from 0 to 337 points. According to the original publication, an HScore of ≥169 points corresponds to a sensitivity of 93% and a specificity of 86% for diagnosing HLH. Notably, several parameters exceeded the upper threshold in most patients, including ferritin, triglycerides, AST, and cytopenias.

Abbreviations: HLH = haemophagocytic lymphohistiocytosis, AST = aspartate aminotransferase, ICU = intensive care unit.

Table S3

Characteristic	Overall<br>N = 378[†]	Trend of parameters before and after treatment initiation<br>-2<br>N = 42[†]	-1<br>N = 42[†]	0<br>N = 42[†]	1<br>N = 42[†]	2<br>N = 42[†]	3<br>N = 42[†]	4<br>N = 42[†]	5<br>N = 42[†]	6<br>N = 42[†]
**Hb (g/dl)**	7.95 (7.47 – 8.79)	7.84 (7.48 – 8.88)	8.23 (7.78 – 8.98)	8.37 (7.96 – 8.79)	8.12 (7.70 – 9.23)	7.82 (7.39 – 8.79)	7.60 (7.21 – 7.95)	7.66 (7.31 – 8.63)	7.94 (7.39 – 9.30)	7.98 (7.46 – 9.04)
available	254	12	18	29	39	37	34	33	26	26
**WBC (10E9/l)**	4 (1 – 8)	4 (1 – 9)	4 (1 – 8)	4 (1 – 9)	5 (1 – 9)	3 (1 – 9)	4 (1 – 7)	4 (1 – 7)	3 (1 – 8)	4 (1 – 7)
available	264	15	22	36	39	36	34	31	26	25
**Platelets (10E9/l)**	38 (20 – 66)	31 (17 – 43)	38 (19 – 62)	36 (21 – 65)	36 (21 – 81)	39 (19 – 67)	38 (17 – 66)	33 (19 – 68)	41 (22 – 67)	41 (19 – 72)
available	267	15	23	36	41	36	34	31	26	25
**Ferritin (µg/l)**	24,530 (8,566 – 54,652)	7,818 (2,777 – 55,326)	19,611 (5,301 – 47,222)	28,052 (14,083 – 97,126)	32,528 (21,435 – 79,516)	25,937 (11,523 – 72,231)	10,680 (3,728 – 39,873)	18,004 (3,759 – 25,347)	26,727 (5,011 – 36,250)	5,844 (3,012 – 26,708)
available	95	4	8	19	19	14	8	11	6	6
**Fibrinogen (g/l)**	2.40 (1.92 – 3.30)	3.07 (2.08 – 3.70)	2.88 (2.08 – 3.70)	2.42 (2.03 – 3.24)	2.24 (1.90 – 2.91)	2.33 (1.90 – 3.70)	2.28 (1.91 – 3.56)	2.31 (1.96 – 3.04)	2.41 (1.91 – 3.16)	2.42 (1.83 – 3.70)
available	199	11	18	23	29	26	27	25	22	18
**AST (U/l)**	350 (104 – 1,052)	194 (38 – 763)	175 (44 – 640)	330 (83 – 1,808)	485 (219 – 1,654)	500 (135 – 1,046)	642 (103 – 1,335)	408 (89 – 1,089)	239 (78 – 703)	214 (111 – 466)
available	200	10	19	26	34	29	28	22	15	17
**Triglycerides (mg/dl)**	328 (244 – 562)	339 (302 – 567)	284 (205 – 415)	290 (221 – 526)	402 (251 – 606)	324 (249 – 487)	448 (243 – 816)	322 (226 – 407)	254 (136 – 435)	494 (230 – 561)
available	108	6	10	20	23	14	10	11	4	10
**LDH (U/l)**	977 (512 – 2,386)	610 (258 – 647)	694 (395 – 1,204)	1,343 (738 – 3,617)	2,131 (907 – 3,457)	942 (659 – 2,435)	933 (512 – 3,895)	630 (340 – 1,987)	642 (450 – 1,684)	697 (452 – 1,411)
available	165	6	16	24	29	25	23	16	11	15
**CRP (mg/l)**	88 (43 – 150)	129 (114 – 179)	110 (63 – 144)	113 (61 – 163)	89 (57 – 170)	103 (51 – 149)	77 (39 – 137)	68 (26 – 103)	52 (21 – 124)	43 (19 – 124)
available	263	14	23	36	39	36	34	30	26	25
**Lactate (mmol/l)**	2.6 (1.6 – 5.2)	2.8 (2.2 – 3.4)	2.7 (2.1 – 3.8)	3.3 (1.7 – 5.5)	3.6 (1.4 – 7.2)	2.6 (1.7 – 4.8)	2.4 (1.5 – 4.9)	2.3 (1.5 – 5.7)	1.9 (1.5 – 3.9)	1.9 (1.5 – 4.3)
available	254	12	18	29	39	37	34	33	26	26
**Noradrenaline (µg/kg/min)**	0.13 (0.02 – 0.35)	0.05 (0.01 – 0.23)	0.11 (0.04 – 0.60)	0.20 (0.08 – 0.73)	0.23 (0.05 – 0.61)	0.18 (0.04 – 0.39)	0.19 (0.01 – 0.35)	0.10 (0.02 – 0.30)	0.06 (0.00 – 0.31)	0.03 (0.00 – 0.20)
available	209	11	15	25	30	29	29	28	22	20
**PF-ratio (mmHg)**	238 (169 – 313)	223 (166 – 295)	223 (167 – 315)	235 (165 – 360)	213 (140 – 270)	234 (139 – 316)	239 (180 – 298)	253 (169 – 307)	295 (183 – 336)	292 (265 – 350)
available	254	12	18	29	39	37	34	33	26	26
**sIL-2R (U/l)**	11,516 (5,457 – 34,553)	11,608 (5,457 – 17,758)	7,762 (7,762 – 7,762)	15,081 (11,516 – 15,582)	8,822 (4,170 – 34,553)	14,966 (6,600 – 43,390)	NA (NA – NA)	8,072 (1,881 – 58,556)	NA (NA – NA)	57,841 (57,841 – 57,841)
available	19	2	1	3	5	4	0	3	0	1
**AaDO2 (mmHg)**	130 (74 – 227)	142 (86 – 191)	140 (84 – 185)	155 (58 – 295)	154 (102 – 365)	139 (84 – 250)	135 (98 – 245)	122 (86 – 192)	89 (57 – 211)	74 (35 – 119)
available	254	12	18	29	39	37	34	33	26	26

[†] Median (IQR)

doi: 10.3389/fmed.2025.1641659

Supplementary Table 3. **Temporal progression of key clinical and laboratory parameters.**

Day 0 represents the date of HLH diagnosis; values are displayed for each parameter from two days before (–2) to six days after (+6) that point. At every time-step the patient-level median (IQR) is shown, and the superscript N indicates how many patients had data available for that variable on the given day.

Abbreviations: HLH = haemophagocytic lymphohistiocytosis, Hb = haemoglobin, WBC = white blood cell count, AST = aspartate aminotransferase, LDH = lactate dehydrogenase, CRP = C-reactive protein, sIL-2R = soluble interleukin-2 receptor, AaDO2 = alveolar-arterial oxygen difference.

Table S4

Characteristic	N	Overall N = 42	ICU Mortality: Survived N = 15	ICU Mortality: Died N = 27	p-value[1]
**Ferritin (µg/l)**	39				**0.039**
Median (Q1-Q3)		23,240.0 (10,599.9-46,502.2)	14,153.7 (7,009.7-22,592.4)	28,060.1 (14,083.0-79,516.1)	
Min, Max		984.5, 393,391.0	984.5, 84,468.6	2,777.8, 393,391.0	
**Fibrinogen (g/l)**	38				0.80
Median (Q1-Q3)		2.4 (1.9-3.3)	2.5 (2.2-3.3)	2.3 (1.9-3.4)	
Min, Max		1.5, 6.6	1.6, 4.0	1.5, 6.6	
**Triglycerides (mg/dl)**	42				0.24
Median (Q1-Q3)		330.5 (251.0-504.0)	287.0 (241.5-358.0)	401.0 (251.0-646.0)	
Min, Max		84.0, 1,426.0	177.0, 567.0	84.0, 1,426.0	
**AST (U/l)**	41				0.13
Median (Q1-Q3)		375.0 (116.3-838.5)	163.5 (93.5-690.0)	500.0 (116.3-1,429.5)	
Min, Max		17.0, 8,993.5	17.0, 1,749.0	35.0, 8,993.5	
**sIL-2R (U/ml)**	12				**0.041**
Median (Q1-Q3)		8,429.3 (5,789.0-28,024.0)	6,941.5 (5,457.0-8,036.5)	28,024.0 (15,081.0-57,841.0)	
Min, Max		1,881.0, 65,489.0	1,881.0, 8,822.0	4,100.0, 65,489.0	
**IL-6 (pg/ml)**	17				0.59
Median (Q1-Q3)		223.4 (47.4-1,231.0)	129.3 (47.4-228.4)	342.0 (36.7-1,329.2)	
Min, Max		10.6, 183,442.6	12.4, 183,442.6	10.6, 8,729.6	

[1] Wilcoxon rank-sum test

Supplementary Table 4. Key laboratory parameters around HLH diagnosis (day 0)

Overall values and stratification by ICU mortality. Data represent the patient-level median for each analyte calculated from daily measurements obtained between two days before (-2) and six days after (+6) the diagnostic date. For each variable, the table lists the cohort median with inter-quartile range (Q1–Q3) as well as minimum and maximum, followed by the same statistics for survivors and non-survivors.

Abbreviations: HLH = haemophagocytic lymphohistiocytosis; AST = aspartate aminotransferase; sIL-2R = soluble interleukin-2 receptor; IL-6 = interleukin-6.

doi: 10.3389/fmed.2025.1641659

Figure S1

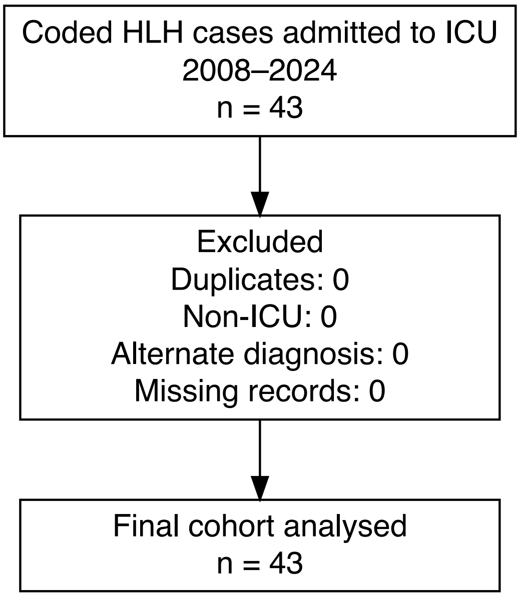

Supplementary Figure 1. Study flow diagram.

Identification of haemophagocytic lymphohistiocytosis (HLH) cases admitted to the intensive care unit (ICU) between 2008 and 2024. All 43 cases identified were eligible and included in the final analysis; no duplicates, non-ICU cases, alternate diagnoses, or missing records were found.

Figure S2

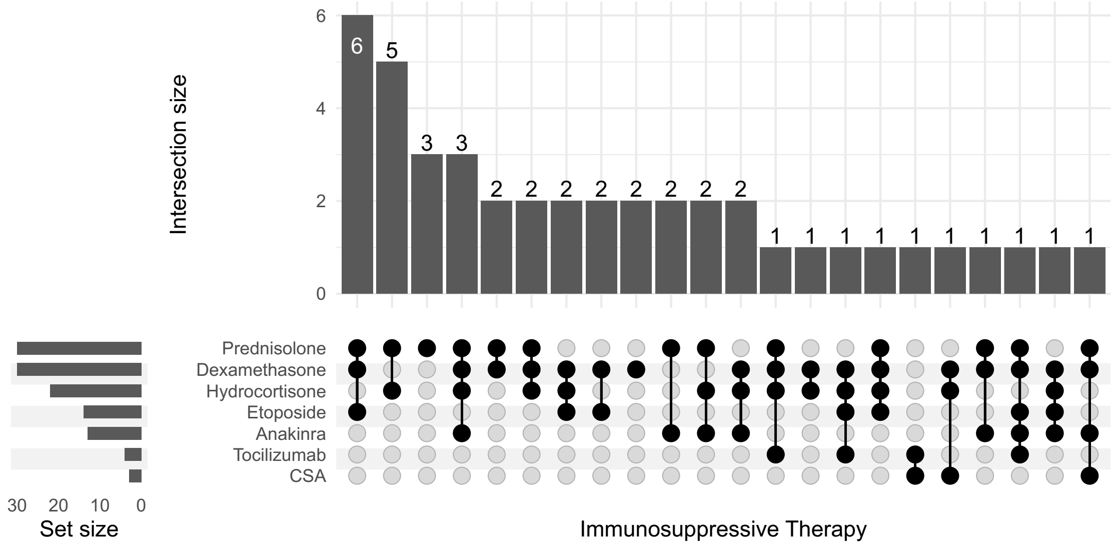

Supplementary Figure 2. Immunosuppressive Therapies

Different combinations of immunosuppressive therapies. The upset chart below highlights how these therapies overlap, with the intersection size representing the number of patients receiving specific combinations and the set size showing how many patients received each individual therapy.

Abbreviations: CSA = ciclosporin A.
